# Trabectedin for Patients with Advanced Soft Tissue Sarcoma: A Non-Interventional, Prospective, Multicenter, Phase IV Trial

**DOI:** 10.3390/cancers14215234

**Published:** 2022-10-25

**Authors:** Viktor Grünwald, Daniel Pink, Gerlinde Egerer, Enrico Schalk, Marinela Augustin, Christoph K. W. Deinzer, Viola Kob, Dietmar Reichert, Maxim Kebenko, Stephan Brandl, Dennis Hahn, Lars H. Lindner, Mathias Hoiczyk, Uta Ringsdorf, Lars C. Hanker, Dirk Hempel, Beatriz De Rivas, Tobias Wismann, Philipp Ivanyi

**Affiliations:** 1 Innere Klinik (Tumorforschung) und Klinik für Urologie, Universitätsklinikum Essen, 45147 Essen, Germany; 2 Helios Klinikum Bad-Saarow, Klinik für Onkologie und Palliativmedizin, Sarkomzentrum Berlin-Brandenburg, 15526 Bad Saarow, Germany; 3 Innere Medizin C, Universitätsmedizin Greifswald, 17489 Greifswald, Germany; 4 Klinik für Hämatologie, Onkologie, Rheumatologie, Universitätsklinikum Heidelberg, 69120 Heidelberg, Germany; 5 Medical Center, Otto-von-Guericke University Magdeburg, 39106 Magdeburg, Germany; 6 Klinikum Nürnberg Nord, Schwerpunkt Onkologie/Hämatologie, Klinik für Innere Medizin 5, Paracelsus Medizinische Privatuniversität, 90419 Nürnberg, Germany; 7 Hämatologie und Internistische Onkologie, Medizinische Klinik und Poliklinik II, Universitätsklinikum Würzburg, 97078 Würzburg, Germany; 8 Schwerpunktpraxis Hämatologie/Onkologie Volksdorf, MVZ für Immunologie Lokstedt GmbH, 20251 Hamburg, Germany; 9 Gemeinschaftspraxis für Hämatologie und Onkologie, Kuhlenstraße 53d, 26655 Westerstede, Germany; 10 Klinik für Hämatologie und Onkologie, Universitätsklinikum Schleswig-Holstein, 23538 Lübeck, Germany; 11 Überörtliche Gemeinschaftspraxis Bergedorf, 21029 Hamburg, Germany; 12 Klinik für Hämatologie, Onkologie und Palliativmedizin, Klinikum Stuttgart, 70174 Stuttgart, Germany; 13 Medizinische Klinik und Poliklinik III, LMU Klinikum-Campus Großhadern, 81377 München, Germany; 14 Medizinischen Klinik II, Marien-Hospital Wesel gGmbH, 46483 Wesel, Germany; 15 Lahn-Dill-Kliniken GmbH, Gynäkologischen Tumorzentrums Lahn-Dill, 35578 Wetzlar, Germany; 16 Klinik für Frauenheilkunde und Geburtshilfe (Gynäkologie), Universitätsklinikum Schleswig-Holstein, 23538 Lübeck, Germany; 17 Onkologisches Zentrum, 86609 Donauwörth, Germany; 18 Medical Affairs, PharmaMar, S.A., Colmenar Viejo, 28770 Madrid, Spain; 19 Praxis für Onkologie und Urologie, 26389 Wilhelmshaven, Germany; 20 Hämostaseologie, Onkologie und Stammzelltransplantation, Klinik für Hämatologie, Claudia von Schilling Zentrum Comprehensive Cancer Center, Medizinische Hochschule Hannover, 30625 Hannover, Germany

**Keywords:** trabectedin, STS, sarcoma, non-interventional, prospective

## Abstract

**Simple Summary:**

Active therapeutic options in advanced soft tissue sarcoma (STS), able to induce durable objective responses, are limited beyond first-line chemotherapy. Although results obtained in clinical trials suggest there is a high probability for patients with STS to benefit from treatment with trabectedin (Yondelis^®^), there is still a paucity of robust real-life data in more diverse patient populations. The prospective, non-interventional phase IV YON-SAR trial (NCT02367924) was designed to evaluate treatment effects of trabectedin in patients with advanced STS in real-life clinical practice across Germany. The efficacy results of this trial, conducted in 128 patients from 19 sites across Germany, further support trabectedin as a standard of care for a second- or further-line treatment of patients with advanced STS in routine clinical practice (median progression-free survival: 5.2 months; median overall survival: 15.2 months). The safety profile of trabectedin was manageable and in line with those observed in previous studies.

**Abstract:**

This non-interventional, prospective phase IV trial evaluated trabectedin in patients with soft tissue sarcoma (STS) in real-life clinical practice across Germany. The primary endpoints were progression-free survival (PFS) rates at 3 and 6 months, as defined by investigators. Overall, 128 patients from 19 German sites were evaluated for efficacy and 130 for safety. Median age was 58.5 years (range: 23–84) and leiomyosarcoma was the most frequent histotype (*n* = 45; 35.2%). Trabectedin was mostly used as second/third-line treatment (*n* = 91; 71.1%). Median PFS was 5.2 months (95% CI: 3.3–6.7), with 60.7% and 44.5% of patients free from progression at 3 and 6 months, respectively. Median overall survival was 15.2 months (95% CI: 9.6–21.4). One patient achieved a complete and 14 patients a partial response, conferring an objective response rate of 11.7%. Decreases in white blood cells (27.0% of patients), platelets (16.2%) and neutrophils (13.1%) and increased alanine aminotransferase (10.8%) were the most common trabectedin-related grade 3/4 adverse drug reactions. Two deaths due to pneumonia and sepsis were considered trabectedin-related. Trabectedin confers clinically meaningful activity in patients with multiple STS histotypes, comparable to that previously observed in clinical trials and other non-interventional studies, and with a manageable safety profile.

## 1. Introduction

Soft tissue sarcomas (STS) are a heterogeneous group of rare malignancies with mesenchymal origin that comprise approximately 1% of adult and 7% of pediatric malignancies [1,2,3]. Once metastatic or unresectable, prognosis for advanced STS is poor, and patients are not considered curable. For patients with advanced STS, systemic chemotherapy has been a cornerstone of treatment, although local therapies such as surgery and radiation therapy may achieve prolonged survival in selected patients [4].

Trabectedin (Yondelis^®^) is an alkylating agent with a multifaceted mechanism of action, which, apart from being a DNA-binding agent, also has selective anti-inflammatory, immunomodulatory and anti-angiogenic properties [5,6,7]. In 2007, trabectedin was the first marine-derived antineoplastic drug approved in the European Union for treatment of patients with advanced STS after failure of anthracyclines and ifosfamide, or who are unsuited to receive these agents [8]. Since 2015, following the analysis of a pivotal, active-controlled, randomized phase III trial in patients with advanced liposarcoma or leiomyosarcoma (commonly abbreviated as L-sarcomas) after failure of prior anthracycline-containing chemotherapy, trabectedin was also approved by the U.S. Food and Drug Administration [9]. Trabectedin was reported to be active in non-L-sarcomas as well since it has demonstrated efficacy in patients with a variety of sarcoma histotypes [10,11,12,13]. In a clinical trial setting, the efficacy of trabectedin compared to best supportive care is also supported by the results of trials conducted in patients with histologically different sarcoma subtypes [14,15]. In addition, trabectedin has a manageable safety profile and is without cumulative toxicities, including those in patients treated for prolonged periods [16].

Although results obtained in clinical trials suggest there is a high probability for patients with STS to benefit from treatment with trabectedin, at the time this study was launched, there was a paucity of robust real-life data on a more diverse patient population than that recruited in clinical trials. Indeed, such observational studies can provide useful insights into the real-life efficacy, safety and management of patients who may be underrepresented in clinical trials due to more restrictive eligibility criteria. Recently, one European and two national observational studies on the real-life use of trabectedin in patients with advanced STS reported clinically meaningful long-term benefits in patients with multiple STS histotypes, largely comparable to those previously reported in selected populations from clinical trials, and with a manageable safety profile [17,18,19].

Currently, data on the real-life use of trabectedin for STS in Germany are limited [20]. The constant change in patient selection and novel therapies applied in advanced STS mandate re-evaluation of real-life management in this evolving treatment landscape. Therefore, the prospective, non-interventional YON-SAR trial (http://www.ClinicalTrials.gov Identifier: NCT02367924) was designed with the aim of evaluating treatment effects of trabectedin in patients with advanced STS across a contemporary treatment landscape in Germany.

## 2. Materials and Methods

This non-interventional, prospective, multicenter phase IV study evaluated trabectedin in routine clinical practice in patients with advanced STS in Germany [21]. Treatment decisions, dosing, monitoring as well as diagnostic or therapeutic procedures were at the discretion of the investigator, were performed according to routine care and were not mandated by the observational plan.

Eligible participants included adults (≥18 years old) with histologically diagnosed advanced STS and who signed an informed consent document. All eligible patients had either progressive disease following therapy with anthracyclines and ifosfamide or were unsuited to receive these agents, and were suitable to undergo treatment with trabectedin according to the Summary of Product Characteristics (SmPC). Patients with contraindications for treatment with trabectedin according to SmPC were excluded.

Trabectedin was administered in agreement with the marketing authorization, standard local clinical practice, and the treating clinician’s discretion. The recommended dose of trabectedin for the treatment of STS is 1.5 mg/m^2^ body surface area, given intravenously over 24 h every 3 weeks. There were no predefined limits of administered trabectedin cycles, and treatment could continue until disease progression, intolerance or consent withdrawal.

The observational period for each patient enrolled in this study consisted of the treatment period and the follow-up period. The treatment period began from the date of the first administration of trabectedin until progressive disease, death or treatment termination (whichever occurred first). After the end of treatment, patients were followed up for at least 12 months. After trabectedin treatment discontinuation, patients could have been treated with subsequent anticancer therapies or supportive care as per the treating clinician’s clinical judgment.

All study procedures were conducted in accordance with the ethical standards as laid down in the 1964 Declaration of Helsinki and its later amendments, guidelines for Good Clinical Practice and local regulations on clinical trials, and were approved by the independent ethics committee.

The primary endpoint of this study was to assess the number of patients free from progression at 3 and 6 months after treatment (i.e., progression-free survival [PFS] rate at 3 and 6 months) as measured by institutional routine clinical standards. Secondary efficacy endpoints included unconfirmed disease control rate (DCR), defined as the percentage of patients with a complete response (CR) or partial response (PR) and/or stable disease (SD), PFS, overall survival (OS) and OS rates at 3 and 6 months after treatment. Secondary endpoints also included an assessment of the treatment with trabectedin and employed doses, treatment duration, causes for treatment discontinuation, and safety profile. The PFS was defined as the time interval from the first administration of trabectedin to the date of disease progression or death, regardless of cause (whichever occurred first), whereas OS was defined as the time between the start of trabectedin and patient death from any cause. Patients not experiencing an event or death or considered lost to follow-up were censored with the date of last contact or with the beginning of the following therapy (whichever occurred first). Response evaluations (i.e., CR, PR and SD) were measured according to local institutional standards, being preferred according to Response Evaluation Criteria in Solid Tumors (RECIST) 1.1 [22] or Choi criteria [23]. Adverse events (AE) were reported according to CTCAE 4.03 and their relationship to trabectedin. Treatment-related AEs were followed until resolution or start of new therapy. Documentation of AEs and serious AEs (SAE) occurred until 30 days after the last dose.

All collected parameters were analyzed in a descriptive manner. Categorical variables were expressed as absolute and relative frequencies, and continuous variables were described by number of observations, median, and range (minimum to maximum). Frequency tables ware prepared for categorical variables and checked for dependencies by Fisher’s exact test. The exact binomial estimator and its 95% confidence interval (CI) were used in the analysis of categorical outcome parameters (e.g., tumor control rate). Time-to-event endpoints (i.e., PFS and OS) and their fixed-time estimations were estimated according to the Kaplan–Meier method and were compared using the log-rank test, while Cox regression models were performed for covariate analyses. All *p*-values were descriptive in nature and the significance level selected was 0.05. All statistical analyses were performed by means of SAS version 9.4 software (SAS Institute Inc., Cary, NC, USA). The efficacy analyses were based on the modified intention-to-treat (mITT) population, defined as all patients who received at least one dose of trabectedin, signed informed consent, and did not violate any inclusion or exclusion criterion. The analysis of safety was performed on the Safety Analysis Set (SAS) that included all enrolled patients who received at least one dose of trabectedin and provided consent to participate in the study.

## 3. Results

### 3.1. Patient Disposition and Characteristics

**Figure 1:**
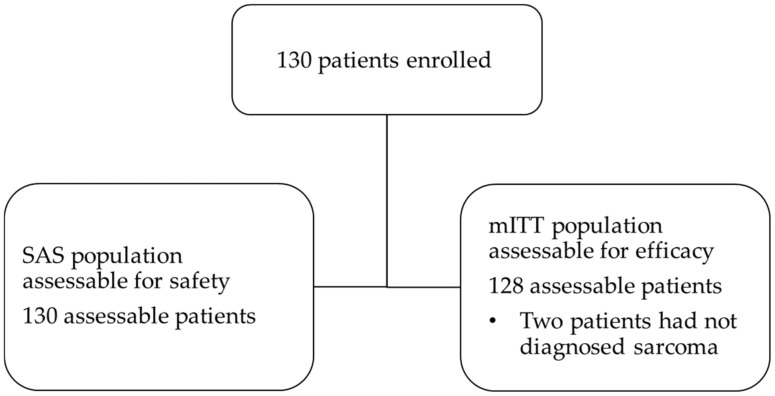
Description of included patients.

Between 16 July 2015 and 22 January 2019, a total of 130 patients from 19 medical centers in Germany were enrolled and received at least one dose of trabectedin (SAS). Two patients were excluded due to lack of STS diagnosis, and therefore, 128 patients were included in the mITT (Figure 1).

The mITT included 65 females (50.8%) and 63 males (49.2%) with a median age of 58.5 years (range: 23–84) (Table 1). An Eastern Cooperative Oncology Group (ECOG) performance status score of 0–1 was recorded in 100 (78.2%) patients. The most prevalent histological type of sarcoma was leiomyosarcoma (*n* = 45, 35.2%), followed by liposarcoma (*n* = 23, 18.0%) and pleomorphic undifferentiated sarcoma (*n* = 20, 15.6%), mostly being localized in lower extremity (*n* = 23, 18.0%), abdomen (retroperitoneal) (*n* = 20, 15.6%), upper extremity (*n* = 17, 13.3%) or uterus (*n* = 15, 11.7%). The most common tumor grade was grade 3 both in patients assessed according to FNCLCC (*n* = 34, 45.3%) and UICC (*n* = 23, 42.6%) system grading, and most had stage IV sarcoma as per AJCC staging system (Table 1). The majority of patients had metastatic disease (*n* = 79, 61.7%), mostly being localized in the lung (*n* = 59, 74.7%), liver (*n* = 25, 31.7%), or bones (*n* = 22, 27.9%).

Previous therapies included surgery in 111 (86.7%) patients, while 64 (50%) patients were treated with radiotherapy. The majority of patients received systemic therapy (*n* = 101; 78.9%), mostly with doxorubicin (*n* = 87, 86.1%) and/or ifosfamide (*n* = 63, 62.4%). Twenty-seven (21.1%) patients were chemotherapy-naïve (Table 2). Most patients received one or two lines of prior systemic therapy (*n* = 91; 71.1%). Overall, 113 patients (88.3%) reported relevant concomitant disease, mainly being arterial hypertension (*n* = 54, 47.8%), other cardiac diseases (*n* = 29, 25.7%) or thyropathy (*n* = 25, 22.1%).

Subsequent antineoplastic therapies were given to 78 (60.8%) patients, which consisted of gemcitabine (*n* = 34, 43.6%), pazopanib (*n* = 31, 39.7%), dacarbazine (*n* = 24, 30.8%) or other treatments (*n* = 33, 42.3%).

### 3.2. Extent of Exposure

Although all patients were suitable to undergo treatment with trabectedin according to SmPC, therapy deviated from the approved label in 67 patients (52.3%), commonly due to a reduced starting dose lower than 1.5 mg/m^2^ (*n* = 53, 41.4%), use of aprepitant as premedication at first cycle (*n* = 14, 10.9%), and lack of baseline biochemistry or hematology in 10 (7.8%) and 5 patients (3.9%), respectively.

Patients received a median of four trabectedin cycles, with 52 (40.6%) patients receiving ≥6 cycles and up to a maximum of 44 cycles (Table 3). Of note, four patients received >24 cycles of treatment (approximately 18 months), one of whom reached 44 cycles of treatment with trabectedin (Table S1). Patients received a median cumulative total dose of 10.7 mg/m^2^ (range: 1.8–110.9) over a median infusion duration of 24 h (range: 3.0–24.2). Premedication consisted of corticosteroids in 96.7% and antiemetics in 75.0% or more patients in each trabectedin cycle. The use of aprepitant was registered in 4.4% to 20.0% of patients during the study.

Dose reductions occurred in 61 patients (47.7%), and dose delays in 76 patients (59.4%). Among 125 (97.7%) patients who discontinued trabectedin, the most frequent reason for treatment discontinuation was progression (*n* = 77, 60.2%), followed by death or other reason (*n* = 12, 9.4% each), patient’s wish (*n* = 10, 7.8%) and trabectedin-related adverse drug reactions (ADRs) (*n* = 9, 7.0%).

### 3.3. Primary Efficacy Endpoint

**Figure 2:**
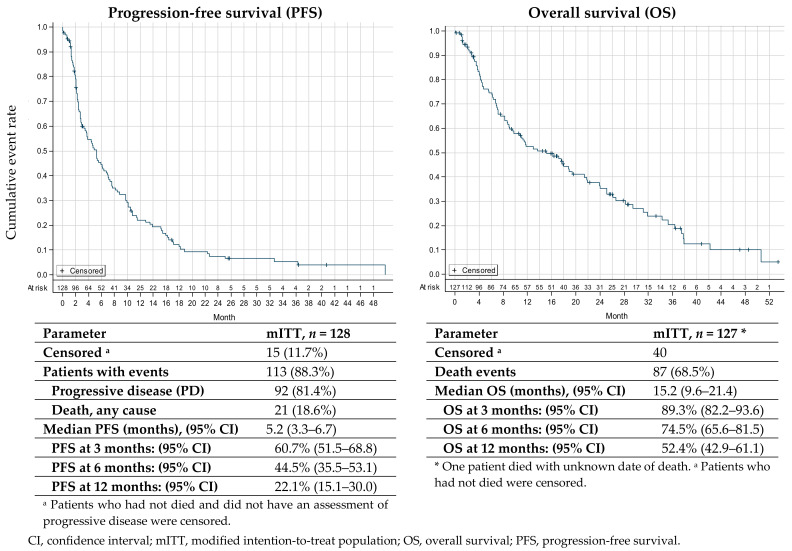
Kaplan–Meier plot of progression-free survival and overall survival.

The analysis of the primary endpoint revealed that 60.7% (95% CI: 51.5–68.8) and 44.5% (95% CI: 35.5–53.1) of patients were free from progression at 3 and 6 months after treatment, respectively (Figure 2).

### 3.4. Secondary Efficacy Endpoint

In the mITT after a median follow-up of 28.7 months (range 0.07–53.5), a total of 113 progression or death events (88.3% of patients) were recorded, whereas 15 (11.7%) patients who were alive or not assessed for disease progression at the time of this analysis were censored. Median PFS was 5.2 months (95% CI: 3.3–6.7) (Figure 2).

Median PFS was similar between patients with reduced dosing during the study (i.e., patients who received at least one trabectedin dose <1.5 mg/m^2^ throughout the study) and in those with reduced starting dose (7.1 months, 95% CI: 4.8–9.8 vs. 5.4 months, 95% CI: 3.7–9.8), as well as in patients fully treated according to the SmPC and those who had treatment deviations from the SmPC recommendations (4.6 months, 95% CI: 2.7–7.1 vs. 5.2 months, 95% CI: 3.3–7.3). Similarly, there were no statistically significant differences in median PFS (*p* = 0.41) among patients who received trabectedin as first- (6.4 months, 95% CI: 2.7–10.8), second (5.3 months, 95% CI: 2.8–7.7), third (4.0 months, 95% CI: 2.0–6.2) and ≥fourth- (3.4 months, 95% CI: 0.7–9.8) line of treatment. After 87 death events (68.5% of patients), treatment with trabectedin resulted in a median OS of 15.2 months (95% CI: 9.6–21.4), with 89.3%, 74.5%, and 52.4% of patients alive 3, 6 and 12 months after treatment (Figure 2), respectively. No statistical difference was detected for OS in patients with reduced dosing during the study (18.9 months, 95% CI: 11.6–25.1) and in those with reduced starting dose (13.7 months, 95% CI: 8.3–26.6) and according to trabectedin treatment line (first-line: 24.0 months, 95% CI: 8.9-not reached; second-line: 15.2 months, 95% CI: 6.9–21.8; third-line: 11.0 months, 95% CI: 6.1–19.4; and ≥fourth-line: 17.5 months, 95% CI: 3.5-not reached; *p* = 0.1558).

Regarding the overall trabectedin activity, one patient (0.8%) had a CR, and 14 (10.9%) patients achieved a PR, reaching the ORR of 11.7%. Additionally, 43 (33.6%) patients had SD as a best result for a DCR of 45.3% (Table 4). Conversely, comparable DCR was observed between patients treated with trabectedin in different treatment lines, and a logistic regression analysis also revealed that presence or absence of tumor metastases at baseline was not statistically associated with DCR outcomes (odds ratio: 0.53, 95% CI: 0.23–1.24, *p* = 0.1421). The ORR and DCR were similar among patients <70 and ≥70 years old (ORR: 12.1% in <70 years and 10.3% in ≥70 years; DCR: 45.4% in <70 years and 41.4% in ≥70 years; post-hoc analysis). Regarding histology, CR was observed in one patient with liposarcoma, and PR was recorded in six patients with liposarcoma, in four patients with other histologies, and in two patients with leiomyosarcoma and synovial sarcoma, while stable disease was observed in patients with several histologies (Table S2).

Additionally, throughout the study, ECOG performance status improved by 1 in 10 (7.8%) patients, deteriorated by 1 in 38 (29.7%), and remained unchanged in 64 (50.0%) patients.

### 3.5. Safety

A total of 86 (66.2%) patients had at least one grade ≥3 AE. Most common (≥10% of patients) grade-3/4 AEs were decreased white blood cell count (*n* = 35, 26.9% of patients), decreased platelet count (*n* = 22, 16.9%), decreased neutrophil count (*n* = 17, 13.1%), increased alanine aminotransferase and anemia (*n* = 14, 10.8% each), and increased gamma-glutamyl transferase (*n* = 13, 10%). Nine (6.9%) patients experienced 10 grade-5 AEs, namely sepsis (*n* = 4, 3.1%), pneumonia, a combination of pneumonia and other infections and infestations, acute coronary syndrome, death not otherwise specified, and benign, malignant and unspecified neoplasm in one patient each (*n* = 1, 0.8% each). Forty-two (32.3%) patients had at least one SAE.

A total of 105 (80.8%) patients had at least one trabectedin-related ADR of any grade, 71 (54.6%) of whom experienced grade ≥3 ADRs (Table 5). Forty-four patients (33.8%) have grade ≥3 ADRs leading to dose modifications. Treatment-emergent serious ADRs (SADR) were uncommon, as a total of 19 (14.6%) patients presented with at least one SADR. No new or unexpected safety concerns were identified for trabectedin.

## 4. Discussion

YON-SAR was the first prospective, multicenter, non-interventional, phase IV study that evaluated trabectedin’s outcomes in routine clinical practice in patients with advanced STS in Germany. While randomized controlled clinical trials are the cornerstone standard of medical evidence, their generalizability to daily clinical practice in a diverse and unselected patient populations always should be verified in non-interventional studies [24]. Of note, considering that in our study we included data from 130 patients from 19 sites across Germany, our data can surely provide a good representation of German real-life clinical practice.

The results of this study corroborate that trabectedin is an active treatment that offers clinical benefits to patients with multiple STS histotypes. In our study, trabectedin administration resulted in a median PFS of 5.2 months with 3- and 6-month PFS rates (primary endpoint) of 60.7% and 44.5%, respectively, which largely exceeded the 3- and 6-month PFS rate thresholds (i.e., 39% and 14%, respectively) established by the EORTC for active agents for the treatment of unselected STS [25] and are either close to or even exceed the new benchmarks proposed only for advanced/metastatic liposarcoma (63% and 44%) and synovial sarcoma (60% and 41%) [26]. However, the nature of our study may limit the interpretation of these observations. Recognizing that direct comparisons cannot be established, the efficacy outcomes of the present study are comparable with the reported median PFS previously reported in phase II (range: 3.3–7.2 months) [8,14,27] and phase III (range: 3.1–4.2 months) trials [9,15]. Furthermore, the results are in line with other observational studies investigating trabectedin in STS (Table 6). In TrObs and RetrospectYon studies, a tendency toward better PFS in patients treated in an early treatment line was demonstrated [18,19]. Although YON-SAR did not observe significant differences in median PFS with respect to treatment lines, PFS estimates indicate that higher PFS may be achieved in earlier lines. Unfortunately, the small sample size of these subgroups is a major limitation and precludes drawing definite conclusions. Conversely, retrospective data from 101 German patients with advanced STS revealed a similar finding [20]. A median PFS of 2.1 months was reported in that study. However, the majority of patients received trabectedin as third or later line (73%). In the fraction of patients who received trabectedin as first or second line, the median PFS was 5.7 months.

YON-SAR reported a median OS of 15.2 months (95% CI: 9.6–21.4). Median OS in this observational study tended to be slightly longer than previously reported in phase II and III trials (12.4–13.9 months) [8,9,15,28] and observational study RetrospectYon (12.2 months) [19]. However, other observational studies reported comparatively longer median OS of 21.3 months and 21.6 months [17,18] (Table 6). This variance in survival is likely explained by differences in patient populations. Several studies of trabectedin indicated that median OS is longer in L-sarcoma patients than in those with other histologies [29]. Conversely, the fraction of liposarcoma patients varied among studies (YON-SAR: 18.0%; Y-IMAGE: 23.4% [17]; TrObs: 30.3% [18]) and, thus, may contribute to differences in outcomes.

In our study, treatment duration was an important factor for long-term outcomes. Patients who received ≥6 trabectedin cycles obtained higher response rates than those who received <6 cycles (Table 3). This observation has been previously reported, and protracted trabectedin treatment beyond 6 cycles is supported both in retrospective [19,30] and prospective series, such as in phase II T-DIS study [26,31]. Clearly, a selection bias applies in this subgroup of patients, and contributing factors other than treatment duration cannot be excluded. However, our data indicate that patients who achieve disease control and tolerate trabectedin treatment can be safely treated beyond 6 cycles, until progression.

Furthermore, in our study nearly 60% of patients reported either improved or unchanged ECOG performance status during the study period. These data could indicate a low disease-related worsening during the treatment with trabectedin, and when the symptoms worsened, this was largely caused by the natural course of disease, since 60% of patients discontinued the treatment due to disease progression.

Although all enrolled patients were suitable to undergo treatment with trabectedin, we observed that therapy deviated from the approved label according to SmPC in 67 patients (52.3%). It is important to note that deviation from SmPC did not affect the efficacy of trabectedin in term of PFS. As per investigator decision, trabectedin was given to 53 (41.4%) patients at a lower dose than that recommended (i.e., 1.5 mg/m^2^). Moreover, in the present study, median PFS and OS were similar between patients treated with reduced starting trabectedin dose and those with reduced dosing during the study; however, comparison of different trabectedin dosages was not the objective of this study. Although our data did not indicate major differences in outcomes among patients with reduced trabectedin doses, the putative effectivity of full-dose trabectedin remains unknown in these patients. This regimen yielded more tumor shrinkage and superior time to progression compared to a weekly regimen. Although we believe it is imperative to use the recommended starting dose of trabectedin and consider treatment modifications only during therapy as specified in the SmPC, our data indicated that in selected cases, and always based on clinical judgment to optimize patient outcomes, reduced doses may be used.

Although aprepitant is not recommended as premedication for trabectedin, 10.9% of patients received aprepitant prior to the first cycle of trabectedin, and 4.4% to 20.0% of patients during therapy. Aprepitant was recognized to potentially increase trabectedin exposure and exert thereby an additional risk of toxicity if given concomitantly [32]. In such cases, close monitoring is required and appropriate dose adjustments should be applied in the event of toxicities.

The safety profile of trabectedin was in line with prior experience and reports, characterized by myelosuppression and hepatic toxicities [16]. In our study, trabectedin demonstrated a favorable safety profile over long-term treatment, as >40% of patients received ≥6 cycles of trabectedin and up to a maximum of 44 cycles of treatment. This is consistent with previous reports where comparable numbers of patients were treated with ≥6 cycles (e.g., RetrospectYon: 34.4%; TrObs: 36.5%; Y-IMAGE: 56.9% of patients) [17,18,19]. 

According to the non-interventional nature of this study, the exact time points and method of response assessment were not previously fixed but were determined according to the clinician’s discretion and with no central radiological review and response confirmation; thus, our data must be interpreted with caution. Moreover, missing or unavailable data can additionally hamper the interpretation of the results. Nevertheless, in spite of these limitations, our real-life study complements well the findings from the clinical trials with trabectedin, as it provides information on unselected patient characteristics treated in routine treatment practices.

## 5. Conclusions

In conclusion, the findings of this non-interventional and prospective phase IV study in Germany consistently support that trabectedin is an active treatment in a routine clinical setting. The overall data observed in our study are in line with those observed in clinical and non-interventional studies and further support the use of trabectedin for patients with multiple sarcoma histotypes.

## Figures and Tables

**Table 1:** Patient and disease characteristics at baseline.

Patients	Modified Intent-to-Treat Set (mITT) ^1^ *n* = 128	
Age at study entry (years)	Median (range)	58.5 (23–84)
	≤60 years	70 (54.7%)
	>60 years	58 (45.3%)
	≤70 years	99 (77.3%)
	>70 years	29 (22.7%)
Gender	Female	65 (50.8%)
	Male	63 (49.2%)
Histology	Leiomyosarcoma	45 (35.2%)
	Liposarcoma	23 (18.0%)
	Pleomorphic undifferentiated sarcoma	20 (15.6%)
	Synovial sarcoma	8 (6.3%)
	Fibrosarcoma	8 (6.3%)
	Angiosarcoma	1 (0.8%)
	Other	23 (18.0%)
Site of primary tumor	Lower extremity	23 (18.0%)
	Abdomen (retroperitoneal)	20 (15.6%)
	Upper extremity	17 (13.3%)
	Uterus	15 (11.7%)
	Abdomen (intraperitoneal)	11 (8.6%)
	Other	42 (32.8%)
Eastern Cooperative Oncology Group (ECOG) performance status	0	34 (26.6%)
	1	66 (51.6%)
	2	10 (7.8%)
	3	1 (0.8%)
	4	1 (0.8%)
	Missing	16 (12.5%)
Tumor grade according to the French Federation of Cancer Centers Sarcoma Group grading systems (FNCLCC) ^2^	Grade 1	13 (17.3%)
	Grade 2	21 (28.0%)
	Grade 3	34 (45.3%)
	Grade X ^3^	6 (8.0%)
	Missing	1 (1.3%)
According to the Union for International Cancer Control (UICC) ^2^	Grade 1	5 (9.3%)
	Grade 2	9 (16.7%)
	Grade 3	23 (42.6%)
	Grade 4	3 (5.6%)
	Grade X ^3^	13 (24.1%)
	Missing	1 (1.9%)
Tumor stage according to the American Joint Committee on Cancer (AJCC)	Ia	3 (2.3)
	Ib	7 (5.5)
	IIa	4 (3.1)
	IIb	5 (3.9)
	III	15 (11.7)
	IV	26 (20.3)
	Unknown	68 (53.1)
Time from first diagnosis to first treatment (months); *n* = 125	Median (range)	0.4 (0.0–149.7)
Time from diagnosis to last treatment before trabectedin (months); *n* = 125	Median (range)	15.9 (0.0–250.2)
Time from last progression to trabectedin treatment (months); *n* = 95	Median (range)	0.9 (0.0–2.5)

^1^ Modified intent-to-treat set (mITT) included all patients who received at least one dose of trabectedin, signed informed consent and did not violate any inclusion or exclusion criterion. ^2^ One patient had no documented grading according to both FNCLCC and UICC and was counted in both grading categories as missing. ^3^ Tumor grade could not be assessed.

**Table 2:** Prior treatments.

Patients	Modified Intent-to-Treat Set (mITT); *n* = 128	
Prior treatments	Prior surgery	111 (86.7%)
	Prior radiotherapy	64 (50.0%)
	Prior chemotherapy/ targeted treatments	101 (78.9%)
No. of lines of prior chemotherapy/targeted treatments, *n* = 128	0 lines	27 (21.1%)
	1 line	66 (51.6%)
	2 lines	25 (19.5%)
	≥3 lines (3 to 6 lines)	10 (7.8%)
Types of prior chemotherapy/targeted treatments, *n* = 101 (≥4% of patients)	Doxorubicin	87 (86.1%)
	Ifosfamide	63 (62.4%)
	Dacarbazine (DTIC)	18 (17.8%)
	Trophosphamide	16 (15.8%)
	Gemcitabine	13 (12.9%)
	Docetaxel	12 (11.9%)
	Epirubicin	12 (11.9%)
	Olaratumab	10 (9.9%)
	Pazopanib	8 (7.9%)
Best response to last prior chemotherapy/targeted treatments, *n* = 101	Complete response (CR)	2 (2.0%)
	Partial response (PR)	22 (21.8%)
	Stable disease (SD)	39 (38.6%)
	Progressive disease (PD)	22 (21.8%)
	Non evaluated (NE)	16 (15.8%)

**Table 3:** Trabectedin exposure.

Treatment Delivery	Modified Intent-to-Treat Set (mITT); *n* = 128	
Number of cycles received per patient	Median (range)	4 (1–44)
	<6 cycles	76 (59.4%)
	≥6 cycles	52 (40.6%)
Dose reductions (per patient)	0 cycle	62 (48.4%)
	1 cycle	17 (13.3%)
	2 cycles	21 (16.4%)
	>2 cycles	18 (14.1%)
	Unknown ^1^	10 (7.8%)
Cycle delays (per patient)	0 cycle	49 (38.3%)
	1 cycle	22 (17.2%)
	2 cycles	8 (6.3%)
	>2 cycles	44 (34.4%)
	Unknown ^1^	5 (3.9%)

Data shown are numbers and percentages of patients or median and range values with available data. ^1^ Patients who started the treatment with trabectedin before the signed informed consents and with unknown starting dose.

**Table 4:** Best response rate according to investigator assessment by number of treatment cycles.

Best Response to Trabectedin per Patient (Unconfirmed)	Modified Intent-to-Treat Set (mITT); *n* = 128		
	<6 Cycles *n* = 76	≥6 Cycles *n* = 52	Total *n* = 128
Complete response (CR)	-	1 (1.9%)	1 (0.8%)
Partial response (PR)	1 (1.3%)	13 (25.0%)	14 (10.9%)
Stable disease (SD)	10 (13.2%)	33 (63.5%)	43 (33.6%)
Progressive disease (PD)	35 (46.1%)	3 (5.8%)	38 (29.7%)
Not evaluable	4 (5.3%)	2 (3.9%)	6 (4.7%)
Not done	26 (34.2%)	-	26 (20.3%)
Objective response rate (ORR; CR + PR)	1 (1.3%)	14 (26.9%)	15 (11.7%)
Disease control rate (DCR; ORR + SD)	11 (14.5%)	47 (89.4%)	58 (45.3%)
Fisher’s exact test (*p*-value) ^1^	<0.0001		-

^1^ Unevaluated patients and those with missing best responses were excluded from the comparison.

**Table 5:** Treatment-related adverse drug reactions (ADRs) in at least ≥3% of patients and all grade-5 ADRs as reported by the investigators (all treated patients).

Treatment-Related ADR as per NCI-CTC, Worst Grade per Patient (≥3% of Patients)	Safety Analysis Set ^1,2^ *n* = 130											
	Grade 1 *n* = 79		Grade 2 *n* = 73		Grade 3 *n* = 67		Grade 4 *n* = 23		Grade 5 *n* = 2		Total *n* = 105	
	*n*	%	*n*	%	*n*	%	*n*	%	*n*	%	*n*	%
ALT increased	9	6.9	11	8.5	14	10.8	-	-	-	-	34	26.2
AP increased	5	3.9	3	2.3	1	0.8	-	-	-	-	9	6.9
Anemia	10	7.7	16	12.3	12	9.2	-	-	-	-	38	29.2
Anorexia	15	11.5	5	3.9	3	2.3	-	-	-	-	23	17.7
Arthralgia	2	1.5	2	1.5			-	-	-	-	4	3.1
AST increased	9	6.9	9	6.9	5	3.9	-	-	-	-	23	17.7
Leukopenia	2	1.5	3	2.3	1	0.8	-	-	-	-	6	4.6
Constipation	16	12.3	2	1.5			-	-	-	-	18	13.9
Diarrhea	4	3.1	4	3.1	2	1.5	-	-	-	-	10	7.69
Dry skin	4	3.1	-	-	-	-	-	-	-	-	4	3.1
Dysgeusia	5	3.85	2	1.5	-	-	-	-	-	-	7	5.4
Dyspnea	3	2.3	2	1.5	-	-	1	0.8	-	-	6	4.6
Edema limbs	3	2.3	-	-	1	0.8	-	-	-	-	4	3.1
Fatigue	24	18.5	19	14.6	3	2.3	-	-	-	-	46	35.4
Febrile neutropenia	-	-	-	-	4	3.1	1	0.8	-	-	5	3.9
Fever	7	5.4	1	0.8					-	-	8	6.2
Night sweating	4	3.1	-	-	-	c	-	-	-	-	4	3.1
GGT increased	7	5.4	3	2.3	11	8.5	1	0.8	-	-	22	16.9
Headache	5	3.9	1	0.8	-	-	-	-	-	-	6	4.6
Hypoalbuminemia	3	2.3	1	0.8	-	-	-	-	-	-	4	3.1
Pneumonia ^3^	-	-	-	-	2	1.5			1	0.8	3	2.3
Mucositis oral	2	1.5	1	0.8	3	2.3	-	-	-	-	6	4.6
Myalgia	3	2.3	1	0.8	1	0.8	-	-	-	-	5	3.9
Nausea	33	25.4	16	12.3	5	3.9	-	-	-	-	54	41.5
Neutrophil count decreased	2	1.5	5	3.9	10	7.7	7	5.4	-	-	24	18.5
Peripheral sensory neuropathy	3	2.3	1	0.8	1	0.8			-	-	5	3.9
Platelet count decreased	14	10.8	5	3.9	13	10.0	8	6.2	-	-	40	30.8
Sepsis ^3^	-	-	-	-	-	-	2	1.5	1	0.8	3	2.3
Vomiting	16	12.3	7	5.4	3	2.3			-	-	26	20.0
White blood cell decreased	7	5.4	12	9.2	27	20.8	8	6.2	-	-	54	41.4

^1^ Safety Analysis Set included all patients who signed informed consent and received at least one dose of trabectedin. ^2^ The percentages relate to the number of patients in the Safety Analysis Set. ^3^ Grade-5 adverse drug reactions. ADR, adverse drug reactions; ALT, alanine aminotransferase; AP, alkaline phosphatase; AST, aspartate aminotransferase; GGT, Gamma-glutamyltransferase; NCI-CTC, National Cancer Institute Common Toxicity Criteria.

**Table 6:** Relevance of the YON-SAR results within the context of trabectedin treatment for recurrent advanced STS.

Median (95% CI)	Advanced Sarcoma	PFS (Months)	PFS-3/6 (%)	OS (Months)	ORR (%)	SD (%)	DCR (%)
Retrospective, Non-Interventional Studies							
French RetrospectYon database Le Cesne et al., 2015 [19]	STS; *n =* 804	4.4 (3.9–4.9)	59.0/40.0	12.2 (11.0–13.3)	16.5	50.1	66.7
	L-sarcoma; *n =* 481	5.7 (4.9–6.5)	64–69.0/NA	15.0 (13.2–16.8)	18.6	54.0	72.6
TrObs study Palmerini et al., 2021 [18]	STS; *n =* 512	5.1 (4.1–6.7)	NA/46.0	21.6 (19.3–25.0)	13.7 (11.2–17.2)	33.0	46.7 (43.2–51.9)
	L-sarcoma; *n =* 348	8.3 (6–10.1)	NA/55.0	25.9 (22.4–33.4)	16.6	37.4	53.4
	non-L-sarcoma; *n =* 164	2.4 (1.8–3.4)	NA/26.0	11.3 (8.1–16.3)	9.0	23.8	32.3
German retrospective study Hoiczyk M et al., 2013 [20]	STS; *n* = 101	2.1	NA	NA	NA	NA	NA
	L-sarcoma; *n =* 46	3.1	51/38	NA	NA	NA	55
	non-L-sarcoma; *n =* 55	1.6	36/16	NA	NA	NA	34
**Prospective, Non-Interventional Studies**							
Y-IMAGE study Buonadonna et al., 2017 [17]	STS; *n =* 218	5.9 (4.9–7.8)	70.0/49.0	21.3 (18.8–24.3)	26.6 (20.9–33)	39.0	65.6 (58.9–71.9)
YON-SAR study Grünwald et al., 2022	STS; *n =* 128	5.2 (3.3–6.7)	60.7/44.5	15.2 (9.6–21.4)	11.7	33.6	45.3

Results of time-to-event endpoints show median and 95% confidence intervals with available data. CI; confidence intervals; DCR, disease control rate; h, hour; L-sarcoma, liposarcoma or leiomyosarcoma; NA, not available; NR, not reached; ORR, objective response rate; OS, overall survival; PFS, progression-free survival; PFS-3/-6, PFS rate at 3/6 months; SD, stable disease; STS, soft tissue sarcoma.

## Data Availability

The data underlying this article will be shared on reasonable request to the corresponding author.

## Supplementary Materials

The following supporting information can be downloaded at: https://www.mdpi.com/article/10.3390/cancers14215234/s1, Table S1. Characteristics of patients treated with prolonged trabectedin treatment (>24 cycles); Table S2. Best responses by patient and disease characteristics at baseline (post hoc analysis).
