# Supplementary material for: Trabectedin for Patients with Advanced Soft Tissue Sarcoma: A Non-Interventional, Prospective, Multicenter, Phase IV Trial
Source: Cancers (Basel). 2022 Oct 25;14(21):5234. doi: 10.3390/cancers14215234 (PMC9653615; doi:10.3390/cancers14215234)
Supplement: Supplementary file 1 [file cancers-14-05234-s001.zip › Table S1.pdf]

Table S1. Characteristic of patients treated with prolonged trabectedin treatment (>24 cycles)

Number of cycles received	Age	Gender	Race	Histology	Site of primary tumor	Metastatic disease	Prior surgery	Prior radiotherapy	Prior chemotherapy	No. of lines of prior chemotherapy	ECOG[2]
44	72	Male	Caucasian	Liposarcoma	Lower extremity	Yes	Yes	Yes	Yes	1	2
34	66	Female	Caucasian	Liposarcoma	Abdomen retroperitoneal	Yes	Yes	Yes	Yes	2	1
34	46	Female	Caucasian	Leiomyosarcoma	Other[1]	Yes	Yes	No	Yes	3	0
27	61	Male	Caucasian	Liposarcoma	Abdomen intraperitoneal	No	Yes	Yes	Yes	1	0

[1]Unknown dedifferentiated uterine leiomyosarcoma. [2]Eastern Cooperative Oncology Group performance status
