# Supplementary material for: Trabectedin for Patients with Advanced Soft Tissue Sarcoma: A Non-Interventional, Prospective, Multicenter, Phase IV Trial
Source: Cancers (Basel). 2022 Oct 25;14(21):5234. doi: 10.3390/cancers14215234 (PMC9653615; doi:10.3390/cancers14215234)
Supplement: Supplementary file 1 [file cancers-14-05234-s001.zip › Table S2.pdf]

Table S2. Best responses by patient and disease characteristics at baseline (Post-hoc analysis)

Patient and disease characteristics at baseline		Modified intent-to-treat set (mITT)[1]; n=128				
		Complete response (CR)	Partial response (PR)	Stable disease (SD)	Progressive disease (PD)	Not evaluable / not done
**Age at study entry (years)**	**N**	1	14	42	38	33
	**Median (Range)**	58.0 (58.0;58.0)	54.50 (28.0;78.0)	58.0 (23.0;84.0)	60.0 (33.0;81.0)	63.0 (26.0;81.0)
	**≤70 Years**	1 (100.0%)	11 (78.6%)	33 (78.6%)	30 (79.0%)	24 (72.7%)
	**>70 Years**	0	3 (21.43%)	9 (21.4%)	8 (21.1%)	9 (27.3%)
**Site of primary tumor**	**Angiosarcoma**	0	0	0	0	1 (3.0%)
	**Fibrosarcoma**	0	0	3 (7.1%)	3 (7.9%)	2 (6.1%)
	**Leiomyosarcoma**	0	2 (14.3%)	18 (42.9%)	13 (34.2%)	12 (36.4%)
	**Liposarcoma**	1 (100.0%)	6 (42.9%)	6 (14.3%)	5 (13.2%)	5 (15.2%)
	**Other**	0	4 (28.6%)	4 (9.5%)	7 (18.4%)	8 (24.2%)
	**Pleomorphic undifferentiated sarcoma**	0	0	7 (16.7%)	10 (26.3%)	3 (9.1%)
	**Synovial sarcoma**	0	2 (14.3%)	4 (9.5%)	0	2 (6.1%)
**Eastern Cooperative Oncology Group (ECOG) performance status**	**0**	0	4 (28.6%)	14 (33.3%)	11 (29.0%)	5 (15.2%)
	**1**	0	8 (57.1%)	21 (50.0%)	22 (57.9%)	15 (45.5%)
	**2**	0	0	4 (9.52%)	4 (10.5%)	2 (6.1%)
	**3**	0	0	0	0	1 (3.0%)
	**4**	0	0	0	0	1 (3.0%)
	**Missing**	1 (100.0%)	2 (14.3%)	3 (7.1%)	1 (2.6%)	9 (27.3%)
	**0-1**	0	12 (85.7%)	35 (83.3%)	33 (86.8%)	20 (60.6%)
	**≥2**	0	0	4 (9.5%)	4 (10.53%)	4 (12.1%)
	**Missing**	1 (100.0%)	2 (14.3%)	3 (7.1%)	1 (2.6%)	9 (27.3%)

[1] Modified intent-to-treat set (mITT) included all patients who received at least one dose of trabectedin, signed informed consent and did not violate any inclusion or exclusion criterion.
